# Processing Mandarin Tone 3 Sandhi at the Morphosyntactic Interface: Reduplication and Lexical Compounds

**DOI:** 10.3389/fpsyg.2021.713665

**Published:** 2021-08-26

**Authors:** Feier Gao, Siqi Lyu, Chien-Jer Charles Lin

**Affiliations:** ^1^Department of Linguistics, Indiana University Bloomington, Bloomington, IN, United States; ^2^Institute of Psychology, University of Tartu, Tartu, Estonia; ^3^Department of East Asian Languages and Cultures, Indiana University Bloomington, Bloomington, IN, United States

**Keywords:** Mandarin tone 3 sandhi, processing, underlying representation, surface representation, reduplication, model of lexical representation

## Abstract

Mandarin tone 3 sandhi is a phonological alternation in which the initial tone 3 (i.e., low tone) syllable changes to a tone 2 (i.e., rising tone) when followed by another tone 3. The present study used a *cross-modal syllable-morpheme matching* experiment to examine how native speakers process the sandhi sequences derived from verb reduplication and compounding, respectively. Embedded in a visually-presented sentential context, a disyllabic sequence containing a sandhi target was displayed simultaneously with a monosyllabic audio, either a tone 1 (i.e., high-level tone), tone 2 (i.e., rising tone) or tone 3 (i.e., low tone), and participants judged whether the audio syllable matched the visual morpheme. Results showed that the tone 3 sandhi was processed differently in the two constructions. The underlying tone and the surface tone were co-activated and competed with each other in sandhi compounds whereas predominant activation of the underlying tone, over the surface tone, was observed in reduplication. The processing of tone 3 sandhi offers support for distinctive morphological structures: a lexical compound is represented both as a whole-word unit and as a combination of two individual morphemes whereas a verb reduplication is represented and accessed as a monomorphemic unit in the mental lexicon.

## Introduction

During spoken word recognition, language users access a word by mapping the speech input to the stored representation. However, acoustic input often deviates from its phonemic representation due to factors such as speech rate, speaker characteristics, co-articulation, and phonological alternation (Weber and Scharenborg, 2012). Morphemes undergoing phonological alternations surface as different allomorphs in the specific phonological environments, and the underlying-surface mismatch thereby creates challenges to morpheme recognition (e.g., *dog[z], cat[s], bus[ɨz]*). Therefore, when processing phonologically alternated sequences in connected speech, the acoustic input itself is often insufficient for spoken word recognition (e.g., Nolan, 1992; Gaskell and Marslen-Wilson, 1996). The current study aims to investigate how native speakers represent and access the underlying and the surface representations at the suprasegmental level—tonal representation in Mandarin Chinese.

### Mandarin Tone 3 Sandhi

Mandarin is a four-way tonal language in which each syllable carries a phonemic tone that distinguishes meanings: Tone 1 is a high-level tone (T1, /*mā*/ “mother”), Tone 2 is a rising tone (T2, /*má*/ “hemp”), T3 is a low tone (T3, /*mǎ*/, “horse”), and Tone 4 is a falling tone (T4, /*mà*/ “scold”). In addition to these four lexical tones, unstressed syllables in Standard Mandarin are referred to as carrying a “neutral tone” (T0; Chao, 1968). The tonal features of a neutralized syllable are not fully realized, as the duration of the neutralized syllable is often shortened, and the pitch is determined by the tone of the preceding syllable (Chao, 1968; Chen, 1999; Duanmu, 2007).

Mandarin T3 sandhi is an example of tonal alternation where a low T3 syllable obligatorily surfaces as a rising tone (T2) when it is followed by another T3 syllable. T3 sandhi leads to a mismatch between the surface and the underlying tone representations as the sandhi word is underlyingly /T3+T3/ but gets realized as [T2+T3] on the surface. This phonological alternation has been attested to consistently apply across different lexical frequency ranges and degrees of lexicality, i.e., occurring in both real and nonce words (Zhang and Lai, 2010), indicating that it is a very productive alternation rule. For example, the compound word *li*-*jie* “to understand” consists of two T3 morphemes, i.e., *li* /T3/ “to notice” and *jie* /T3/ “to solve” where the lexical tone of the first morpheme *li* changes from T3 to T2 as it is followed by another T3 syllable (i.e., *li-jie* /**T3**+T3/ → [**T2**+T3] “to understand”). In addition to lexical compounds, T3 sandhi can also occur in reduplicative structure. When the base morpheme carries a T3, e.g., *xiang* /T3/ “to think,” T3 sandhi is derived after the base reduplicates and the initial/base morpheme changes from T3 to sandhi T2, e.g., *xiang* /T3/ → T2+T3 → *xiang-xiang* [T2+T0] “to think for a little while.”

T3 sandhi poses important processing questions to theories of lexical access and representation for Chinese words. A disyllabic T3 sandhi sequence creates a mismatch between the underlying and surface representations, as the sandhi syllable is underlyingly T3 but gets realized as T2 in the surface representation. This phenomenon raises questions of how native speakers access the underlying and the surface forms during online processing, and how disyllabic words that involve T3 sandhi are stored and accessed in the mental lexicon. As T3 sandhi is a phonological alternation that occurs at the lexical level, we need to review the theories of lexical access and representation of Chinese complex words before moving onto the processing of T3 sandhi.

### Morphological Representation of Mandarin Chinese

In Mandarin Chinese, about 70% of the words are disyllabic (Duanmu, 2000), mostly compounds made of two free morphemes (Li and Thompson, 1981). The dominance of the compounding structure and the salience of the individual morphemes make Mandarin Chinese an interesting case for studies of word recognition. There are two major views central to the representation of compound words in Mandarin Chinese: (1) the single-layer morphemic model, which postulates that polymorphemic Chinese words are represented as a combination of separate morphemes in the mental lexicon, and (2) the two-layer representation model, which postulates that both the whole word and the individual morphemes are represented and accessed.

Early work of Zhang and Peng (1992) represented a morpheme-based approach for processing Mandarin Chinese, arguing that Chinese words are stored in a morphologically decomposed form in the mental lexicon. They conducted a series of visual lexical decision experiments on coordinative (e.g., *fu-xiong* “father and elder brother”) and modifier-noun compounds (e.g., *mu-xiao* mother-school “alma mater”). A positive character frequency effect was found for both constituent positions in coordinative compounds whereas only for the second constituent (the stem) in modifier-noun compounds. Zhang and Peng (1992) argued that the explicit morphological structure is represented in the mental lexicon and the polymorphemic word is accessed through the word's stem. Because both morphemes function as heads in coordinative compounds, they were equally activated in the lexical access. In modifier-noun compounds, the second constituent played a dominant role over the first constituent, and the structural information facilitated the stem activation but inhibited the modifier activation.

Later studies employed priming tasks to further probe the structural influence on Chinese compound word recognition. For example, Ji and Gagné (2007) conducted a visual-visual priming lexical decision tasks on modifier-noun compounds, in which disyllabic primes and targets varied on the semantic relations between the modifiers and the nouns. They found a facilitatory effect when the prime and target matched on their semantic relations both on modifiers (e.g., *shu*-*dia*n “bookstore” and *shu*-*jia* “bookcase”) and on nouns (e.g., *bing-**dian* “cookie store” and *ri-**dian* “day store”). They also found that the facilitation was lost when target head appeared 350 ms before the whole compound was presented, whereas such prolonged exposure to the modifier did not lead to the loss of facilitation. Ji and Gagné thus concluded that the prolonged display of the modifier cannot promote the pattern of relation priming, whereas the increased exposure to the head noun increases the relation priming associated with it. They attributed the results to the stronger role for head noun in the processing of modifier-noun compounds in Chinese. These outlined studies pointed to the decompositionality of Chinese words, emphasizing the role of morphological structure in lexical processing.

On the other hand, disyllabic word meaning is not always fully predictable from the compositionality of individual morphemes. Therefore, it is necessary to also consider a whole-word level of representation regarding lexical access in Chinese. Zhou and Marslen-Wilson (1994, 1995) proposed a *Multi-level Cluster Representation Model*, which assumed that both the whole-word lexical entries and the explicit morphological structure are represented in the mental lexicon. This model argues against the single-layer, morpheme-based approach and utilizes both the lexicalist representations and the morpheme-based combinatory account regarding the lexical access for Chinese compounds. Two following studies further instantiated this two-layer model.

Zhou and Marslen-Wilson (1994) used an auditory lexical decision task to examine the morphological processing of Chinese disyllabic real words (compounds) and non-words. The whole-word, morpheme and syllable frequencies of the 1st or 2nd constituent of the compound were systematically manipulated in three experiments. Their results, overall, showed that word frequency plays a dominant role in spoken word recognition of real compounds and this effect does not interact with either the morpheme or syllable frequency of either the 1st or the 2nd constituent. The observed word frequency effect suggested that the whole word unit is the more salient representation level than the morphemic or the syllabic level during the compound processing in Chinese. In addition, they found that compound processing involves decomposing a word into its morphemes, as the syllable frequency of the 1st constituent showed an effect of slowing down the lexical decision time in both real compounds and non-words. Zhou and Marslen-Wilson (1994) thus concluded that Chinese disyllabic compounds are represented both at the word and morphemic levels, which allows the whole-word lexical unit to be analyzed syllable by syllable as the word is heard.

Zhou and Marslen-Wilson (1995) further conducted a series of auditory-auditory repetition priming tasks in five disyllabic prime-target relationships. In the identical condition, the prime and the target were identical (e.g., *ju*-*ben* “play script” vs. *ju*-*ben* “play script”); in the morphological condition, the prime and the target shared the same morpheme (e.g., *ju*-*chang* “theater” vs. *ju*-*ben* “play script”); in the homophonic condition, the prime and the target shared the same syllable representations but not the same morphemes (e.g., *ju*-*pa* “fear” vs. *ju*-*ben* “play script”); in the homographic condition, the prime and the target shared the same Chinese characters but not the same morphemes (e.g., *ju*-*lie* “violent” vs. *ju*-*ben* “play script”); and the baseline condition in which the prime and the target were unrelated (e.g., *chuang-li* “originate” vs. *ju-ben* “play script”). Constituent positions (i.e., either the 1st or 2nd constituent of the compound) were systematically manipulated in both the primes and the targets. They found a facilitatory priming effect (relative to the baseline condition) in the identical and the morphological condition irrespective of the critical morpheme's position, but the morphological priming size was reduced when the prime and the target matched on their first constituents as opposed to on the second ones. Zhou and Marslen-Wilson concluded that the repeated access to the same morpheme shared between primes and targets facilitated the compound recognition in the morphological condition. When matched on the first constituents, the prime and target words formed cohort members on the word level (e.g., *ju*-*chang* “theater” vs. *ju*-*ben* “play script”) and the cohort competition weakened the morpheme-level facilitatory effect. In the homophonic and homographic conditions, a facilitatory priming effect was found when the prime and target matched on their second constituents, an inhibitory effect when matched on their first constituents, and a null effect when the second constituent of the prime and the first constituent of the target matched. Zhou and Marslen-Wilson attributed the inhibitory effect to the word-level cohort competition between words sharing the homophonic initial syllables, the facilitatory effect to the pre-activation of the homophonic second morpheme without the presence of word-level cohort competition, and the null effect to the cancellation between the word-level competition and the morpheme-level facilitation. Zhou and Marslen-Wilson (1994, 1995) thus proposed a two-layered lexical representation model for investigation on disyllabic Chinese words, combining the morpheme and the whole-word representations in the mental lexicon.

While most previous studies focused on the representation and access of Chinese compounds, the morphological construction beyond compounding has received limited attention. Verb reduplication, a productive morphological process in Standard Mandarin, provides an interesting case for probing the morphological representation in Mandarin Chinese. In Standard Mandarin, verb reduplication adds a sense of casualness to the base verb, meaning to do something “a little bit” or “for a little while” (Li and Thompson, 1981; Tsao, 2001; Xiao and McEnery, 2004). For instance, the monosyllabic verb *ting* /T1/ “to listen to” can be reduplicated to mean “to listen for a little while,” as in *ting-ting* “to listen-RED.” In the disyllabic verb reduplication, the monosyllabic base maintains a lexical tone (e.g., T1 in the first syllable of *ting*-*ting*) while the tone of the reduplicant syllable obligatorily neutralizes to T0 (e.g., the second syllable in *ting-**ting* carries T0). Disyllabic verb reduplication presents a different morphological structure from compounding, as the latter is created through the combination of two individual morphemes while the former is derived by reduplicating a monomorphemic word (base).

Some studies have put forward a syntactic account for representing Mandarin verb reduplication. They argue that the reduplicant syllable is not lexically encoded but serves as an affix (Li and Sui, 2009; Sui, 2018) and the reduplicated full form constitutes a morphological construction above the word level (Arcodia et al., 2014; Sui and Hu, 2016; Basciano and Melloni, 2017; Xie, 2020). In terms of morphological representation, while previous studies found that both morpheme and whole word play a salient role in the lexical access of compound words (Zhou and Marslen-Wilson, 1994, 1995), it remains unknown how disyllabic verb reduplication is represented in the mental lexicon and whether a reduplicated verb is accessed as a disyllabic unit or a monomorphemic word during online processing. In addition, when the two morphological constructions—lexical compounds and verb reduplication—both involve T3 sandhi, the morpho-phonological interaction further poses questions of whether tone sandhi sequences derived from two distinctive morphological processes are represented and accessed differently during online processing.

Disyllabic compounds in Mandarin Chinese undergo T3 sandhi when both morphemes are T3 syllables. Tonal mismatch is created between the underlying and surface levels of representation, as the initial syllable is realized as an underlying T3 at the morphemic level but a sandhi-surfaced T2 at the word level. In the Mandarin verb reduplication that is inflected from a T3 base morpheme, T3 sandhi applies on the initial syllable while the second syllable is further reduced to T0 due to tone neutralization. For example, the monosyllabic base verb *xiang* /T3/ “to think” reduplicates to *xiang-xiang* “to think a little while,” with the following derivation of tones: /T3/ + RED → /T3/ + /T3/ → [T2] + [T3] → [T2+T0]. The T3 sandhi pattern suggests that T3 is realized on the reduplicant, therefore triggering T3 sandhi before it is neutralized (Packard, 1998; Xu, 2001; Sui, 2018). The initial syllable is underlyingly T3 at both the morphemic and word levels but surfaces as a sandhi T2 in the disyllabic reduplicated form. In verb reduplication, tone neutralization gives rise to an opaque surface sequence [T2+T0] where the original sandhi environment (i.e., a T3 – T3 sequence) is lost in the phonetic output. While most previous studies have exclusively investigated the processing of T3 sandhi within the word level (i.e., compound), few studies, to our knowledge, have examined how the reduplication-derived sandhi sequence is processed.

### Tone 3 Sandhi Processing

Regarding the representation of tones in words that involve tone sandhi, three major views have been examined by previous studies: the canonical representation view, the surface representation view, and the underspecification (i.e., abstract representation) view. The canonical representation view takes the citation T3 as the underlyingly stored representation of the sandhi syllable in the mental lexicon. The surface representation view takes the surface T2 as the stored form that is directly accessed during online processing. The underspecification view postulates that the tonal representation of the sandhi syllable abstracts away from specific tones; thereby both T2 and T3 audios can be active and mapped onto the sandhi syllable.

Zhou and Marslen-Wilson (1997) investigated these representation views by conducting an auditory-auditory priming lexical decision task. In their study, a disyllabic tone sandhi target (e.g., *cai-qu* /T3+T3/ → [T2+T3] “adopt”) was preceded by either a disyllabic prime with a T2-initial morpheme (e.g., *cai-hua* /T2+T2/ and [T2+T2] “talent”), a disyllabic prime with a T3-initial morpheme (e.g., *cai*-*hong* /T3+T2/ and [T3+T2] “rainbow”), or a control prime (e.g., *tian*-*e* /T1+T2/ “swan”). The first syllable in the T2 prime condition matched the sandhi syllable on the surface tone (i.e., T2), the first syllable in the T3 prime condition matched on the canonical tone (i.e., T3), and the first syllable in the control prime (i.e., T1) is unrelated to either the surface or the canonical tone. Their results showed that the T3-initial prime facilitated the lexical decision times for the sandhi target, whereas the T2-intial prime slowed down the recognition for the sandhi target. Zhou and Marslen-Wilson (1997) argued that the T3-initial prime pre-activated all canonical T3 morphemes and T3-initial words, thus facilitating the spoken word recognition of the sandhi target. They attributed the inhibition in the T2 prime condition to the word-level cohort competition between the co-activated sandhi-surfaced T2-initial words, canonical T2-initial words and T2 morphemes. These results were interpreted as supporting the surface representation view. In another priming experiment, Zhou and Marslen-Wilson (1997) used sandhi words as one of the prime conditions rather than the target. In this experiment, a disyllabic target word with a T2-initial morpheme (e.g., /*cai-pan*/ /T2-T4/ “referee”) was preceded by a disyllabic prime with either a T2-initial morpheme (e.g., /*cai-chan*/ /T2+T3/ “property”), a T3-initial morpheme (e.g., /*cai-na*/ /T3+T4/ “adopt”), a sandhi-initial syllable (e.g., *cai-fang* /T3+T3/ → [T2+T3] “gather material”), or a control prime (e.g., *yu-liao* /T4+T4/ “predict”). An inhibitory effect on the target word was found for each prime condition, suggesting that canonical T2s, sandhi-surfaced T2s, and canonical T3s could all create lexical competition with words containing a T2-initial morpheme. Zhou and Marslen-Wilson (1997) thus concluded that they could not make a definite choice between the surface and the canonical representation view, since neither of them can accommodate the data from the two experiments. The third view – the underspecification representation view, according to Zhou and Marslen-Wilson, is less likely to account for T3 sandhi word representation. It is not only because neither of the experiments could support this view, but also due to the theoretical concern that underspecifying tonal information in Mandarin may lead to greater morphemic ambiguity and thus higher level of competition and less efficiency during lexical access and word recognition.

More recently, Chien et al. (2016) conducted an auditory-auditory priming lexical decision task. In their experiment, monosyllabic instead of disyllabic primes were used to avoid the influence of the second syllables as shown in Zhou and Marslen-Wilson (1997). Each disyllabic tone sandhi target (e.g., *fu*-*dao* /T3+T3/ → [T2+T3] “to counsel”) was preceded by either a monosyllabic T3 prime (e.g., *fu* /T3/ “to guide”), a monosyllabic T2 prime (e.g., *fu* /T2/ “to assist”), or a monosyllabic control prime (e.g., *fu* /T1/ “to put on”). The pre-activation of a T3 prime was found to facilitate the recognition of a sandhi target word, compared with the control prime and the T2 prime, and there was no inhibitory or facilitatory priming effect found in the T2 prime condition. According to Chien et al., the sandhi syllable is represented as the canonical T3 and the speech input of a T3 syllable therefore facilitates the recognition of the sandhi words, supporting the canonical representation view. These results were partially consistent with the findings in Zhou and Marslen-Wilson (1997), in the sense that a facilitatory effect was found in the T3 prime condition, though the pre-activation of a T2 prime was not found to inhibit word recognition in Chien et al. (2016). This study indicated the important role of underlying representation during lexical access, arguing that the disyllabic sandhi word is represented and accessed in its canonical form /T3+T3/ in the mental lexicon.

Among a series of priming experiments in Meng et al. (2021), two cross-modal *semantic* priming tasks were conducted to examine the role of canonical and surface tone in sandhi word representation. In their first experiment, the disyllabic sandhi targets (e.g., *da-sao* /T3+T3/ → [T2+T3] “to clean”) were visually presented and preceded by one of three monosyllabic audio primes that were minimally contrasted with the sandhi syllable on tones (e.g., *da* /T3/ “to beat,” *da* /T2/ “to answer” or *da* /T4/ “big”). In their second experiment, the same set of audio primes were used, but the visual targets were only semantically related to the sandhi targets from the last experiment (e.g., *qing-li* /T1+T3/ “to clean up”). They found that both the T2 and T3 audio primes (e.g., *da* /T3/ “to beat” and *da* /T2/ “to answer”) could activate and facilitate the sandhi words as well as the semantically mediated words. Meng et al. (2021) contradicted Chien et al. (2016) in terms of the surface tone representation, and they attributed the difference to two possible reasons. First, the lack of T2 priming effect in Chien et al. (2016) could be due to an inhibition effect rather than a lack of facilitation, since the T2 prime and the sandhi syllable were homophonous. Second, Meng et al. (2021) used a cross-modal paradigm with no interval between the prime and target presentation, whereas Chien et al. (2016) used an audio-audio priming task in which the target was played 250 ms after the offset of the prime. The shorter interval in the former study may thus lead to greater tonal activation than in the latter.

While all three studies found a facilitatory priming effect in the T3 prime condition, they diverged in terms of T2 priming effects. The T2 prime was found to inhibit the recognition of the sandhi targets in Zhou and Marslen-Wilson (1997), facilitate it in Meng et al. (2021), and have a null effect in Chien et al. (2016). In summary, these studies consistently found that the underlying tone is activated and accessible in sandhi processing but the surface tonal representation is less clear in terms of its priming effect.

Previous studies investigating the lexical access and representation of T3 sandhi have exclusively focused on the sandhi that occurs within compound words. Few studies, if any, have taken into account morphological structures other than compounding. While it has been found that spoken word recognition can be affected by internal morphological structures such as headedness (e.g., Zhang and Peng, 1992), we suspect that the processing of T3 sandhi within different types of morphological processes such as compounding and reduplication should also exhibit different patterns.

## The Present Study

In the present study, we compare the processing of T3 sandhi that occurs within two structures: lexical compounding and reduplication of a monosyllabic verb stem. The lexical compounds (hereafter referred to as T3-COM) are formed by combining two underlyingly T3 morphemes, where the first syllable undergoes T3 sandhi and the second syllable remains T3 in the surface form (e.g., *li-jie* /T3+T3/ → [T2+T3] “to understand”). In the reduplication of a T3 base morpheme (hereafter referred to as T3-RED), the first syllable undergoes sandhi and surfaces as T2 whereas the tone of the reduplicant (i.e., the second syllable of the sequence) gets neutralized to T0 (i.e., *xiang* /T3/ “to think” → *xiang-xiang* [T2+T0] “to think for a little while”). The comparison between T3-RED and T3-COM allows us to probe the interaction between phonological and morphological representations—how T3 sandhi is processed in compounds and reduplication. The orderings of the tonal alternations are provided in Table 1.

**Table 1:** Tonal derivations of verb reduplication and lexical compounds.

	**T2-RED**	**T3-RED**	**T3-COM**
Underlying form	/T2/ (+ T2)	/T3/ (+ T3)	/T3 + T3/
Tone sandhi	✘	**T2** - T3	**T2** - T3
Tone Neutralization	T2 - **T0**	**T2** - **T0**	✘
Surface form	[T2 - **T0**]	[**T2** - **T0**]	[**T2** - T3]
**Example**	*tan*T2 → *tan*T2-***tan*****T0**	*xiang*T3 → ***xiang*****T2**-***xiang*****T0**	*li*T3*-jie*T3 → ***li*****T2**-*jie*T3
	“to talk for a little while”	“to think for a little while”	“to understand”

*Syllables that undergo tonal alternations are boldfaced and underlined*.

In addition, we included disyllabic non-sandhi verb reduplication inflected from a monosyllabic T2 base verb (hereafter T2-RED), which has identical morphological structure as T3-RED except that it does not involve T3 sandhi (i.e., *tan* /T2/ “to talk” → *tan-tan* [T2-T0] “to talk for a little while”). Both T2-RED and T3-RED are produced as [T2-T0] in the speech output, but only T3-RED involves T3 sandhi. Compared with T3-RED, in which the first syllable is underlyingly T3 but surfaces as T2 in the phonetic form, T2-RED carries a more transparent output where the first constituent is a T2 syllable in both the underlying and surface forms.

The inclusion of T2-RED allows us to compare it with T3-RED regarding the processing difference between non-sandhi and sandhi reduplications. Based on the canonical representation view of spoken word recognition, a “re-writing rule”, also known as phonological inference, must apply to mediate the deviant surface form and the underlying form in the context where phonological alternation can take place. In terms of Mandarin T3 sandhi, the surface T2 is expected to be co-activated in the sandhi context, since it is the phonetic realization of the sandhi syllable. Language users “rewrite” the surface T2 into the underlying T3 so that they can map the audio input to the underlying tonal representation (Pulman and Hepple, 1993; Gaskell and Marslen-Wilson, 1996, 1998). Recent studies provided evidence that the sandhi construction does require additional phonological processing effort compared to the non-sandhi construction (Zhang et al., 2015). Native speakers need to resolve the underlying-surface tone competition in the sandhi construction but not in the non-sandhi context (which does not involve tonal alternations). By investigating sandhi processing in a reduplicated structure, we hope to examine how phonological inference works in an opaque sandhi context where the second T3 syllable is neutralized as T0 in the phonetic output (i.e., T3-RED).

The research goal of our study is thus twofold. First, we investigate the processing of Mandarin T3 sandhi in two different morphological processes—reduplication and compounding (T3-RED vs. T3-COM), probing whether morphological structures influence the representation and access of T3 sandhi sequences. Second, we contrast the processing differences between sandhi and non-sandhi syllables in a reduplicated structure (T2-RED vs. T3-RED). The present study thus hopes to extend the previous findings on T3 sandhi processing by also considering morphological processing of Mandarin Chinese.

## Methods

A *cross-modal syllable-morpheme matching experiment* was conducted where the visual target word was presented in a sentence to match against an audio syllable. A monosyllabic audio, either a T1, a T2, or a T3, was played at the onset of the disyllabic visual target word. Participants were asked to make decision as to whether the audio stimuli matched the initial syllable of the visual target word. Three target constructions for the visual target word were: (1) a reduplicated sandhi verb T3-RED, (2) a compound sandhi verb T3-COM, and (3) a reduplicated non-sandhi verb T2-RED.

### Participants

Thirty-two native Standard Mandarin speakers (22 females, 10 males; mean age of 25.28 years [range, 19–36], SD = 3.80) participated in the experiment and we included 30 of them in our data analysis. As for the two participants that we excluded: one is from Guangdong where Cantonese is the dominant language spoken, and the other is from Taiwan where the Mandarin variety is influenced by Taiwanese Southern Min and other Southern dialects. These two participants self-reported that they speak dialects (Cantonese and Hakka) in addition to Mandarin. As noticed by the experimenter, these two subjects were also unable to produce the neutral T0 even when prompted after the experiment. All of the remaining participants self-reported that they speak Standard Mandarin (*Putonghua*) as their dominant language and find themselves to be more comfortable with speaking *Putonghua* than other dialects. Of these 30 participants, 20 are from Northern China and self-reported speaking Mandarin and Northern Mandarin dialects, 5 are from Central China (transitional regions between Northern and Southern China) and speak Mandarin and Mandarin subdialects, 1 is from Chengdu, Sichuan and speaks Mandarin and Southwestern Mandarin dialect, 2 are from Shanghai and speak Mandarin and some Shanghainese, 2 self-reported migration history across two or more dialectal regions before adulthood and speak Mandarin as their primary language. The experimenter did not notice any obvious accent from them during personal interaction. This study received IRB approval, and each participant gave verbal consent and received a $10 cash payment for participation.

### Stimuli

To construct visual stimuli for target words, 15 underlyingly /T3+T3/ verb compounds, 15 T3 monosyllabic verbs, and 15 T2 monosyllabic verbs were selected based on the following principles. First, the monosyllabic verbs are able to be reduplicated for the delimitative meaning. Second, the initial syllables can be legitimately combined with T1, T2, and T3 so that these auditory stimuli are all real words in Mandarin. Third, to control for the internal morphological structure of the compounds, all the compound words bear a coordinative structure, such as *yan-jiang* /T3+T3/ 演讲 “to give a speech”, which is made of two verb morphemes *yan* T3 演 “to perform” and *jiang* T3 讲 “to talk”. The frequencies of monosyllabic base morpheme of reduplication, initial morpheme of disyllabic compound, and disyllabic full form (whole word frequency of compound and reduplicated form frequency of reduplication) were retrieved from *The Chinese Web Corpus zhTenTen 2017* (accessed *via Sketch Engine* https://www.sketchengine.eu/), a family of Chinese corpora (with 13.5-million-word size) built from Internet texts. Full list of stimuli is provided in Appendix 1.

To avoid homophonic as well as homographic ambiguity associated with the target items, each visual target was presented in a carrier sentence and was visually presented in Chinese characters. Presenting the target words in sentences provides syntactic and semantic contexts for interpreting the target words. The target words appear in the final position of the carrier sentences, which had the same structure in all items. Visually, the first character (target syllable) of the target word was coded in red color to indicate that it was the syllable that the audio input was supposed to represent. All sentences consisted of 10 Chinese characters.

For each sentence, a monosyllabic audio stimulus was played at the onset of the target word presentation for participants to match against the red-coded character. The audio stimuli were recorded by a female native speaker of Standard Mandarin in a sound-proof room using a vocal microphone at a sampling rate of 44100 Hz and later segmented in Praat (Boersma and Weenink, 2013). Each visual target word was paired with one of the three monosyllabic audio files: a T3 syllable, a T2 syllable and a T1 syllable, which were segmentally identical with the initial morpheme and differed only on tones. The audio stimuli were presented in a Latin-square design, such that each participant would see a target word and hear one of its three corresponding sound files only once. The syllable (homophone) frequency of each audio file was retrieved from the online *Cncorpus* 国家语委现代汉语语料库 (with 12.8-million-word size, accessed *via* http://corpus.zhonghuayuwen.org/).^1^ Considering that audio durations may be different between tonal categories and thus affect participants' reaction times, we measured the whole-syllable and rhyme durations of each audio stimulus and included them as predicting factors in the statistical analyses. The descriptive statistics of the frequencies and durations are given in Table 2 below.

**Table 2:** Morpheme, full-form and syllable frequencies and durations.

		**T2-RED**	**T3-RED**	**T3-COM**
		**Mean (sd)**	**Mean (sd)**	**Mean (sd)**
Morpheme frequency		4.191 (4.921)	11.807 (20.775)	5.789 (7.936)
Full frequency		0.172 (0.273)	0.229 (0.654)	2.813 (3.168)
Syllable frequency	T1	63.573 (174.320)	15.541 (23.829)	20.424 (35.986)
	T2	17.035 (19.356)	51.375 (93.327)	32.034 (44.791)
	T3	23.566 (52.982)	27.357 (38.111)	29.507 (47.329)
**Audio**		**T1 mean (sd)**	**T2 mean (sd)**	**T3 mean (sd)**
Syllable duration (s)		0.469 (0.069)	0.500 (0.062)	0.487 (0.058)
Rhyme duration (s)		0.374 (0.037)	0.408 (0.037)	0.396 (0.033)

We analyzed several additional factors to rule out potential confounds on the stimuli, including stroke counts of the initial character and both characters, cloze probability of the target word in the sentential context (i.e., whether participants could predict the final target word from the sentential context), and whole-sentence naturalness scores.^2^ Two-sample *t*-tests were conducted for each variable in two comparisons: T3-RED vs. T3-COM and T2-RED vs. T3-RED. The only variable that was found to be significantly different between the constructions was the naturalness scores of T3-RED and T3-COM (see Table 3). These results indicated that verb reduplication appearing at the sentence-final position was judged as less natural than lexical compounds appearing in the same position. In case this effect has an impact on participants' reaction times or responses, we included it as a predicting factor in our statistical analysis.

**Table 3:** Two-sample *t*-tests (T2-RED vs. T3-RED; T3-RED vs. T3-COM).

	**T2-RED**	**T3-RED**	**T3-COM**	***t*-value 1**	***t*-value 2**
	**Mean (sd)**	**Mean (sd)**	**Mean (sd)**		
Initial stroke	10.800 (3.802)	9.600 (3.225)	8.467 (3.021)	0.932	0.993
Total stroke	21.6 (7.604)	19.200 (6.450)	17.400 (4.881)	0.932	0.862
Cloze probability	0.063 (0.123)	0.047 (0.071)	0.078 (0.200)	0.428	−0.573
Naturalness	39.990 (5.691)	41.141 (5.393)	57.974 (7.096)	−0.568	−7.315^***^

*t value 1 is the result of the T2-RED vs. T3-RED, t value 2 is that of T3-RED vs. T3-COM*.

*The gray-shaded values indicate t-test significance*.

*** *p < 0.001*.

To disguise our research focus, we also included 100 additional disyllabic non-sandhi units presented in carrier sentences as filler words (see Appendix 2). The audio stimuli used for these fillers were segmentally identical to those of the initial character of the filler words (also coded in red color) but might contrast in tone (T1: *n* = 30, T2: *n* = 20, T3: *n* = 22, T4: *n* = 28).

### Procedure

The *cross-modal syllable-morpheme matching* experiment was programmed on E-Prime 2.0 (Schneider et al., 2002) and run on a desktop computer connected to a response box. The visual stimuli were presented using black Simsun fonts (size 72pt) on a white background, and the audio files were played to participants over Sennheiser PC350 headphones. The experiment was conducted in the Language and Cognition Laboratory at Indiana University Bloomington.

**Figure 1:**
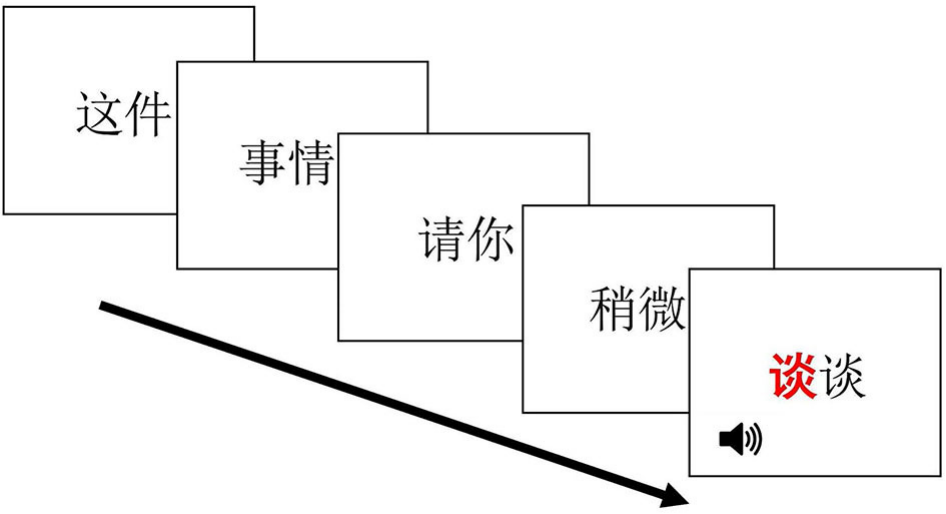
RSVP Experiment Demo (left to right: zhe-jian “this,” shi-qing “thing,” qing-ni “please,” shao-wei “a little bit,” tan-tan “to talk”).

Prior to the experiment, each participant completed a background questionnaire self-reporting their age and language background information. In the instructions, participants were told that they would read a full sentence presented to them every two characters at a time (in 5 separate windows) and an audio file would randomly be played during one of the windows (Figure 1). In each trial, an audio recording of a monosyllabic word began playing as soon as the response word (i.e., target word or filler word) displayed on the screen. Participants were instructed to make decisions on whether the audio stimuli matched the sound of the red-coded character in the response word by pressing either *Yes* or *No* on the response box (marked in Chinese characters, respectively) as quickly as possible. Participants did 10 practice trials before proceeding to the main experiment session. Each trial began with a fixation cross at the center of the screen. Subjects were instructed to press any key to start the trial. The sentences were presented using the *rapid serial visual presentation* (RSVP) paradigm in 5 consecutive windows, and each window lasted 700 ms before being replaced by the next window. Response words were embedded at either the 2nd, 3rd, 4th, or the last window in a pseudo-random order, so that subjects did not know where to expect its occurrence. Proportions of the response words occurring at final and non-final position were balanced (49.7 vs. 50.3%). A blank screen appeared at the end of each trial and lasted for 1,300 ms before next trial started.

Response words in the target sentences was always embedded at the sentence-final position, which ensured that participants were provided enough sentential context before the target, and that no upcoming window would interrupt completion of the matching task. Different from the target sentences, response words in the filler sentences appeared in various positions of the sentence to disguise our research focus. To make sure that participants paid attention to the content of the sentences during the experiment, half of the filler sentences were followed by a binary comprehension question, which appeared on a separate screen following the last window of the sentence. Participants pressed the corresponding *Yes* or *No* key to answer the question. Once an answer was made, the current trial was terminated, and the program automatically proceeded to the next trial without the 1,300 ms blank screen. The total duration of the experiment was around 30–40 min. Participants' syllable-morpheme mapping responses (*Yes*/*No*) and reaction times (RTs) were recorded for analysis. RTs were measured from the onset of the target words to the time of key pressing.

## Results

The mean accuracy for answering the comprehension questions was 86.9% (SD = 0.05) for the remaining 30 participants. RTs longer than 2,000 ms were excluded, accounting for 4% of the total number of observations. Mixed-effects models were fitted to response types (Yes/No) and RTs, respectively, using lme4 package version 1.1–26 (Bates et al., 2015) in R version 4.0.3 (R Core Team, 2020). Response types were analyzed as a binomial dependent variable using logistic linear mixed effects model, and RTs, log-transformed to stabilize variance and ensure normally distributed residuals (Box and Cox, 1964), were analyzed using linear mixed effects models. *Participant* and *Item* were included as random variables. The lmerTest package version 3.1–3 (Kuznetsova et al., 2017) in R was used for obtaining the significance levels. We first present the results for response types, followed by those for RTs.

### Response Types

**Figure 2:**
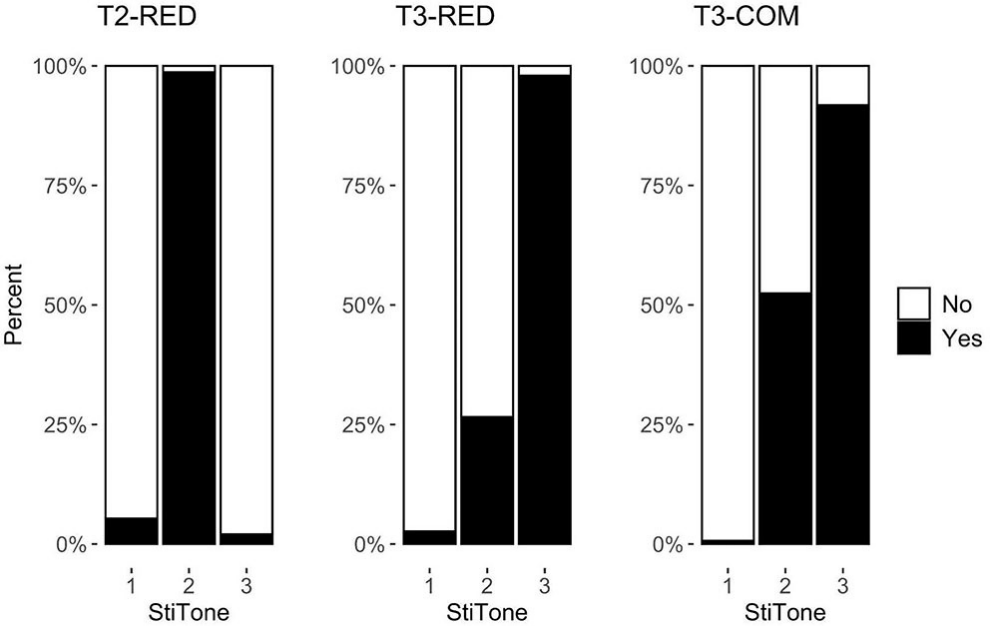
Response types across three target constructions (StiTone refers to the tone of the auditory stimulus item).

Percentages of response types for each construction are given in Figure 2. Recall that T2-RED is inflected from a monosyllabic T2 base verb (*tan* /T2/ → *tan-tan* [T2-T0] “to talk for a little while”), T3-RED is inflected from a monosyllabic T3 base verb (e.g., *xiang* /T3/ → *xiang*-*xiang* [T2-T0] “to think for a little while”), and T3-COM is the compounding of two T3 syllables (e.g., *li-jie* /T3+T3/ → *li*-*jie* [T2+T3] “to understand”). A logistic linear mixed effects model^3^ with a Construction^*^Tone by-participant and *Tone by-item* random slope was constructed first, and a backward stepwise fitting was conducted to select the best fitting model based on Likelihood ratio test significance. A random-slope model^4^ was finally selected as the best fitting model, which contains an interaction between *Construction* and *Tone*, as well as the *Tone by participant* random slope, in predicting the results of response types.

Pairwise comparisons with a Bonferroni correction, using the *emmeans* package version 1.6.1 (Lenth, 2021) in R, were conducted to assess the response differences. The percentages of *Yes* responses to the audio files that matched the underlying tones reached ceiling in all three constructions—T2 in T2-RED (98.7%), T3 in T3-RED (97.9%), and T3 in T3-COM (91.8%), suggesting that participants were highly accurate in mapping the target syllable to the underlying tonal representations. It provided evidence that during speech processing, the underlying representation of the target syllable, whether it is in a sandhi or a non-sandhi sequence, is overwhelmingly taken as the target representation. Despite the ceiling accuracy in all three constructions, *post-hoc* tests showed that the responses given to the underlying tones were significantly different between T2-RED and T3-COM (β = −2.045, *t* = −2.544, *p* < 0.05) but not between any other two constructions (*p*s > 0.05). In contrast, the *Yes* responses given to the T1 audio were consistently low in all three constructions (5.3% in T2-RED, 2.7% in T3-RED, and 0.7% in T3-COM). The floor acceptance rates suggested that participants were able to reject the T1 audio across different conditions. *Post-hoc* tests also showed that the responses were not different among the three constructions (*p*s > 0.05).

We also found that the percentage of *Yes* responses given to the audio that matched the surface tone of sandhi syllable (i.e., T2) were different between sandhi reduplication (i.e., T3-RED) and sandhi compounds (i.e., T3-COM). Participants gave more *Yes* responses to the surface T2 audio in sandhi compounds than in sandhi reduplication (26.6% in T3-RED vs. 52.4% in T3-COM; β = 2.176, *t* = 5.244, adjusted *p* < 0.001). It suggested that, despite the ceiling acceptance of the underlying tone in all constructions, participants were less consistent with the surface tonal representation of a compound than that of a reduplication.

In summary, our results showed that participants could successfully map the target syllable to its underlying representation, regardless of whether it is in a sandhi construction (i.e., T3-RED and T3-COM) or a non-sandhi one (i.e., T2-RED). By contrast, participants were confused in mapping the sandhi syllable to its surface representation. More specifically, they were more inclined to reject the T2 in reduplication than in compounds. As for sandhi compounds, the higher acceptance rate given to the surface tone was in line with the relatively lower acceptance rate for the underlying tone, in comparison with sandhi reduplication. This result suggested that the underlying and the surface representations were more likely to compete with each other in lexical compounds, as opposed to reduplication.

### Reaction Times

The mean log-transformed RTs and standard deviations (sd) of each condition (audio type + construction type + response type) are provided in Table 4 below.

**Table 4:** Mean RTs and SDs in each condition.

**Audio**	**T2-RED**		**T3-RED**		**T3-COM**	
	**Mean (sd)**		**Mean (sd)**		**Mean (SD)**	
	**Yes**	**No**	**Yes**	**No**	**Yes**	**No**
T1	6.598 (0.436)	6.630 (0.226)	6.777 (0.303)	6.693 (0.218)	6.645 (NA)	6.636 (0.202)
T2	6.678 (0.248)	6.423 (0.117)	6.845 (0.329)	6.775 (0.273)	6.795 (0.299)	6.828 (0.230)
T3	6.807 (0.445)	6.697 (0.241)	6.730 (0.245)	7.349 (0.189)	6.700 (0.233)	7.010 (0.364)

*Standard deviation in responding Yes to T1 in T3-COM is not applicable (N = 1)*.

The remaining results are divided into two subsections. The comparison between non-sandhi and sandhi reduplication–T2-RED and T3-RED–is presented first, followed by the comparison between the two morphological structures–T3-RED and T3-COM.

#### Sandhi vs. Non-sandhi: T3-RED vs. T2-RED

To investigate the processing of sandhi vs. non-sandhi sequences, we focus on two reduplicated conditions: T2-RED, which inflected from a T2 base and surfaces as [T2+T0] in the output, and T3-RED, which inflected from a T3 base and surfaces as [T2+T0] as well. Note that the first syllable in T2-RED does not undergo tone sandhi so the underlying and the surface representations are identical (both are T2). Hence, rejecting the T2 audio as well as accepting any audio syllables other than T2 should be considered incorrect (which was confirmed by the floor acceptance rates of T1 and T3 for T2-RED in Figure 2). For our analysis, we specifically focused on the RT data of the *Yes* responses to T2 in T2-RED and the *Yes* responses to T3 in T3-RED, which represent decisions of accepting the underlying representations. Linear mixed effects model were fitted to the RTs in T2-RED and T3-RED, with the following factors included as the fixed predictors (continuous predictors were z-scored):

- **Construction**: categorical predictor with levels T2-RED (reference level) and T3-RED, sum coded.
- **Morpheme frequency**: continuous predictor based on the morpheme frequency of the first character (i.e., red-coded) in each visual target.
- **Full-form frequency**: full-form frequency of each visual target (i.e., the whole-word frequency of T3-COM and the reduplicated-form frequency of T3-RED).
- **Syllable frequency**: homophone frequency of each audio file.
- **Syllable duration**: duration (ms) measured from syllable onset to offset of each audio file.
- **Rhyme duration**: duration (ms) measured from rhyme onset to syllable offset of each audio file.

In this linear mixed effects model, we also included three two-way interactions: Morpheme Frequency^*^Construction, Full Frequency^*^Construction, and Syllable Frequency^*^Construction. We first constructed a full *Construction by-participant* random-slope model containing all candidate predictors^5^ and then employed backward stepwise model selection. The full model was first compared to a random-intercept-only model, and we found that the more complex model was overfitted (χ^2^ = 0.638, *df* = 2, *p* = 0.727). Then, starting from the most complex predictors (i.e., two-way interactions) to the main effects, predictors were eliminated if they did not improve model fitting based on the likelihood ratio test significance. If a simpler model is not tested to be significantly different from a more complex one (*p* > 0.05), we choose the simpler model and continue eliminating more predictors until a significance is generated from model comparison. The final model^6^ output (Table 5) is given in Table 5 below.

**Table 5:** Best fitting model for T2-RED vs. T3-RED comparison.

	**Estimate**	**Std. Error**	**df**	***t* value**	**Pr(>|t|)**
(Intercept)	6.70061	0.02847	33.61248	235.355	<2e-16^***^
construction1	0.10551	0.02984	263.52270	3.537	0.000479^***^
MorFreq	−0.03544	0.01782	272.40455	−1.988	0.047759^*^
SyllFreq	−0.00777	0.04465	271.48153	−0.174	0.861994
construction1:SyllFreq	0.22649	0.08658	266.35174	2.616	0.009408^**^

* *p < 0.05;*

** *p < 0.01;*

*** *p < 0.001*.

Based on the output in Table 5, we found a significant main effect of *Construction*, indicating that the underlying tone of the sandhi construction T3-RED was more effortful to process than that of the non-sandhi construction T2-RED. The latency in T3-RED, compared with T2-RED, was likely to be triggered by the competition between the underlying T3 and the co-activated sandhi-surfaced T2. It suggested that native speakers “rewrote” the surface T2 into the canonical tone even in an opaque context. While participants had to decide between the underlying and surface forms in T3-RED for making a mapping decision, the syllable-morpheme matching was transparent and more straightforward in T2-RED since it involved no tonal alternation. This result thereby supports the canonical representation view.

**Figure 3:**
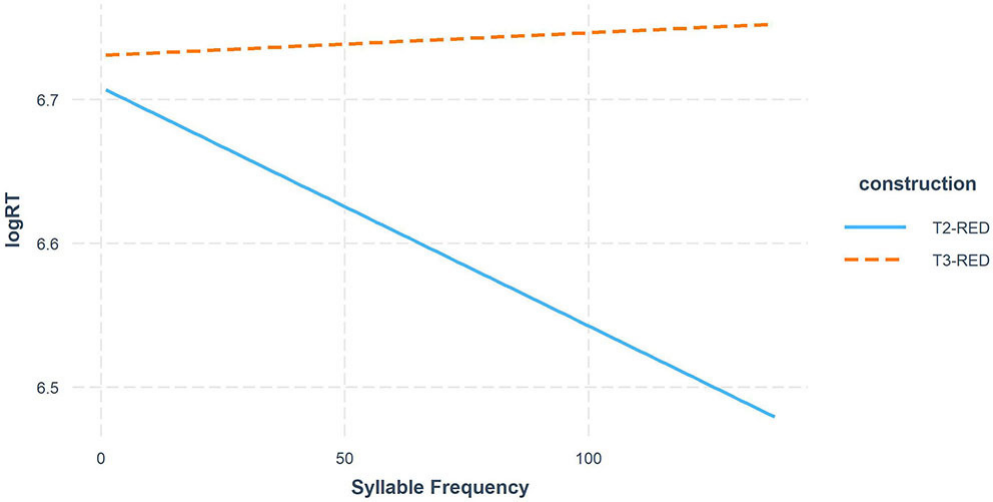
Syllable Frequency effect in T2-RED vs. T3-RED comparison.

We also found a significant interaction between *Construction* and *Syllable Frequency*, with results showing that the frequency effects were in opposite directions: in T2-RED, participants tended to accept the (underlying) tone faster when the (underlying) syllable frequency was higher (β = −0.011, *t* = −1.303, *p* = 0.194), whereas in T3-RED, they tended to take longer time to accept the underlying tone as the underlying syllable frequency increased (β = 0.012, *t* = 0.791, *p* = 0.43). This is likely because in T3-RED, the higher the input syllable (T3) frequency, the more frequently it may show up as a sandhi tone, and thereby the tonal competition between T3 and sandhi T2 is greater. This result is also in line with the main effect of *Construction*, further providing evidence for the canonical representation view. In contrast, higher syllable frequencies facilitated the syllable-morpheme mapping in T2-RED since there was no tonal competition involved. See Figure 3 for the interaction plot.

In addition, higher morpheme frequency shortened the RTs regardless of construction type, as participants accepted the underlying tones significantly faster when the target morpheme frequency was higher. This result is interesting in the sense that, as opposed to the tonal competition at the phonological level, the surface tone did not seem to interfere with the processing at morphemic level. The inclusion of the full-form frequency was not found to improve the model fitting therefore it was removed in the final model.

Taken together, although both surfacing as [T2+T0] in the phonetic form, sandhi reduplication showed a distinct processing pattern from a non-sandhi one. Both the main effect of *Construction* and the *Syllable Frequency* interaction suggested that processing the sandhi reduplication was more effortful than the non-sandhi one, likely because language users had to sort through the canonical representation and the co-activated surface representation in the sandhi construction. These patterns suggested that native speakers still parse the T3-RED as a sandhi-undergoing construction even though it surfaces in an opaque output.

#### Reduplication vs. Compounding: T3-RED vs. T3-COM

Motivated by our research question of how T3 sandhi is processed in compounds and reduplication, we fitted mixed effects linear regression models to the RT data of processing T3-RED and T3-COM (where the underlying tone is T3 and the surface tone is T2). In this mixed effects model, *Construction* (T3-RED and T3-COM, sum coded with T3-RED as the reference level) is the variable of interest. In addition to the predictors mentioned in the last subsection, we also included *Naturalness*, which was tested to be significantly different between T3-RED and T3-COM. To take into account the potential response effect reported in Response Types, we coded the response-audio combinations with four categorical levels: “yes-underlying”, meaning that the participants gave a *Yes* response to the underlying T3 audio, “no-underlying”, meaning that the participants gave a *No* response to the T3, “yes-surface”, meaning that the participants gave a *Yes* response to the surface T2 audio, and “no-surface”, meaning that the participants gave a *No* response to the T2. We named this 4-level variable as *Decision* and included it as a predictor in our model. Because T1 was irrelevant to either the underlying or the surface form of the sandhi syllable and it was rejected in both constructions as we expected, we excluded the T1 data from the RT analysis.

We included several interactions that we are interested in. A two-way interaction between *Construction* and *Decision* was included, allowing us to examine differences in sandhi processing between compounds and reduplication with the response effect taken into account. We also included a series of three-way interactions, *Morpheme*/*Full*/Syllable Frequency^*^Construction^*^Decision, to assess whether morphemic-, full-form- and phonological-level frequency, respectively, modulates the T3 sandhi processing in different constructions (motivated by *Multi-level Cluster Representation Model* analysis by Zhou and Marslen-Wilson, 1994, 1995). Following the same backward model selection as we conducted in Response Types and Sandhi vs. Non-sandhi: T3-RED vs. T2-RED, we constructed a full model^7^ with a Construction^*^Decision by-participant and *Decision by-item* random slope first and predictors each eliminated in a stepwise fashion if they did not significantly improve model fitting. The final model^8^ output is included in Table 6 below.

**Table 6:** Linear mixed effects regression model: T3-RED vs. T3-COM.

	**Estimate**	**Std. Error**	**df**	***t* value**	**Pr(>|t|)**
(Intercept)	6.682546	0.029827	46.49497	224.0443	<2e-16^***^
Decisionyes-surface	0.095379	0.028837	574.2961	3.307535	0.001000^**^
Decisionno-underlying	0.433249	0.082829	566.0419	5.230656	2.38e-07^***^
Decisionno-surface	0.085368	0.023446	558.7801	3.641098	0.000297^***^
construction1	0.003755	0.042135	173.645	0.089118	0.929091
MorFreq	0.013347	0.015426	72.22392	0.865224	0.389781
FullFreq	−0.04898	0.034962	46.5351	−1.40088	0.167884
Decisionyes-surface:construction1	−0.02284	0.055373	541.2572	−0.41255	0.6801
Decisionno-underlying:construction1	−0.34667	0.157713	549.2381	−2.19809	0.028359^*^
Decisionno-surface:construction1	0.084684	0.047077	538.234	1.798829	0.072606.
Decisionyes-surface:MorFreq	−0.0125	0.023962	547.8875	−0.52148	0.602247
Decisionno-underlying:MorFreq	0.279218	0.109363	556.1608	2.553126	0.010942^*^
Decisionno-surface:MorFreq	0.011093	0.018877	542.1806	0.587612	0.557037
Construction1:FullFreq	0.162761	0.073361	29.44491	2.218623	0.034374^*^

. *p < 0.1;*

* *p < 0.05;*

** *p < 0.01;*

*** *p < 0.001*.

We found a significant main effect of *Decision*, as well as two-way interactions of Decision^*^Construction, Morpheme Frequency^*^Decision, and Full Frequency^*^Construction on RTs. The inclusion of the syllable frequency did not improve the model fitting; therefore it was removed in the final model. Our data, overall, showed that T3 sandhi was processed differently in compounds and reduplication and that the multi-level frequency effects participated unequally during processing.

**Figure 4:**
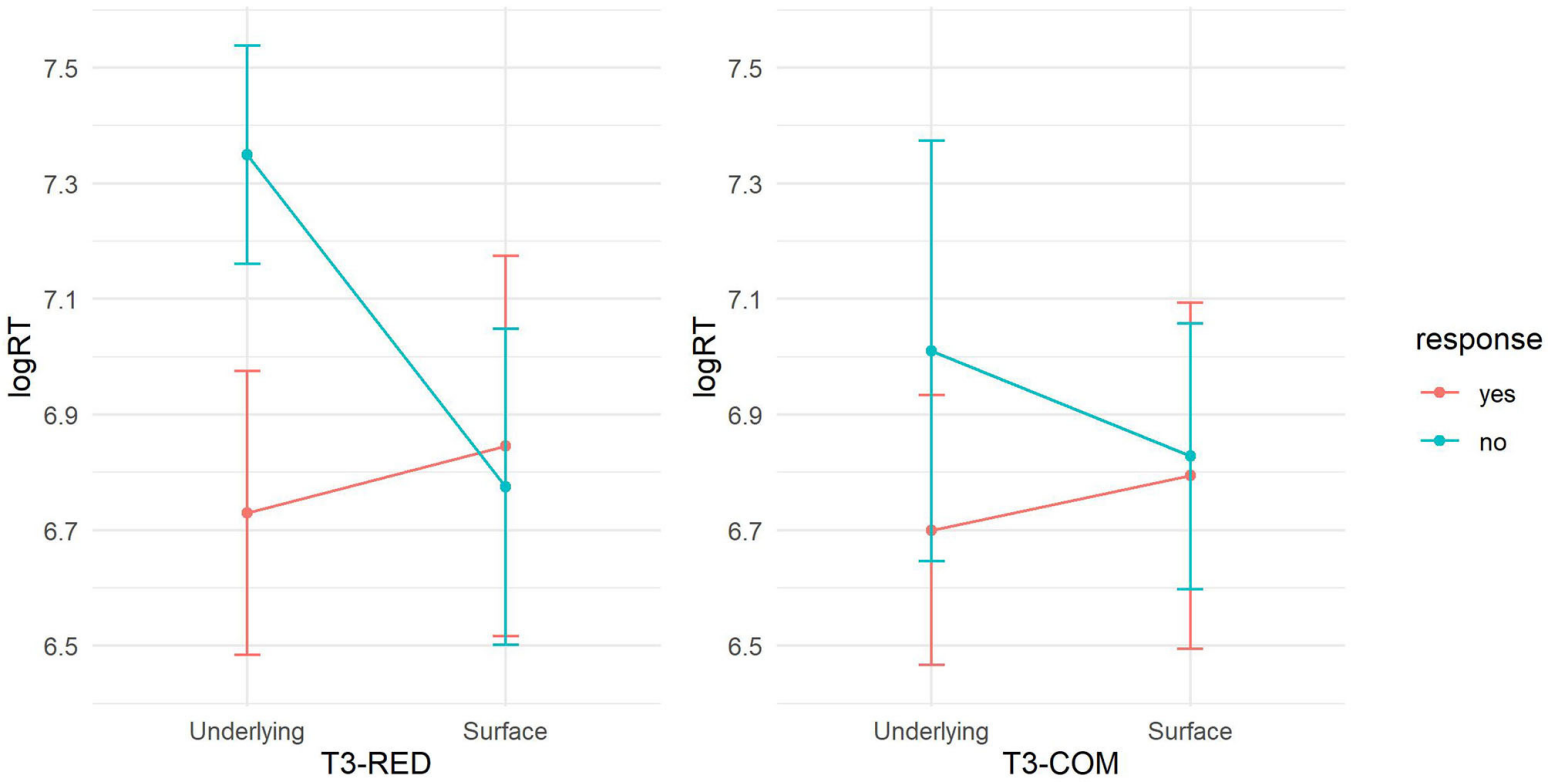
RTs between construction and response types.

Pairwise comparisons with a Bonferroni correction, using the *emmeans* package version 1.6.1 (Lenth, 2021), were conducted to further examine the significant two-way interaction between Decision^*^Construction, broken down by construction type, i.e., T3-RED and T3-COM. In both constructions, “no-underlying” elicited longer RTs than did “yes-underlying” (T3-RED: β = 0.642, *t* = 4.262, *p* < 0.001; T3-COM: β = 0.295, *t* = 3.825, *p* < 0.001) but with a greater magnitude in T3-RED than in T3-COM (T3-RED: *p* = 0.0001; T3-COM: *p* = 0.0009), suggesting that rejecting the underlying representation was harder than accepting it and this difference was greater in reduplication. We found that “no-surface” was not significantly different from “yes-surface” in both constructions (*p*s > 0.05). This pattern indicated that participants were confused whether the T2 audio matched the sandhi syllable, irrespective of construction. Also, for both constructions, the differences between “yes-surface” and “yes-underlying” were non-significant (*p*s > 0.05), suggesting that efforts of accepting the surface tone and the underlying tone were similar between reduplication and compounds. The RT difference between “no-surface” and “no-underlying” was significant in T3-RED (β = −0.597, *t* = −3.946, *p* < 0.001) but *not* in T3-COM (β = −0.166, *t* = −2.074, *p* > 0.05), suggesting that the efforts between rejecting the surface tone and the underlying tone were different in reduplication but *not* in compounds. See Figure 4 for RT visualization.

These results suggested that in both constructions, (1) rejecting the underlying T3 was harder than accepting it, and (2) participants were confused at parsing the sandhi syllable as its surface T2. These patterns suggested an overall dominant effect of underlying tone representation in T3 sandhi processing, as participants were inclined to access the sandhi syllable as a T3 rather than a T2, irrespective of construction. Moreover, we observed an easier access to the surface tone in sandhi compounds than in sandhi reduplication, suggesting a stronger role of the surface representation during the tonal mapping in the former than in the latter.

The interaction of Morpheme Frequency^*^Decision suggested that the frequency effect at the morphemic level was significantly different across decision types. Based on the model output in Table 6, the morpheme frequency effect was significant in “no-underlying” as compared with the baseline “yes-underlying,” suggesting that the RT difference between rejecting and accepting the underlying tone increases as the morpheme frequency increases. We then switched the baseline to “no-underlying” and further found that the frequency effect in “no-surface” was significant (β = −0.268, *t* = −2.447, *p* < 0.05), indicating that the RT difference between rejecting the surface tone and the underlying tone decreases as the frequency increases. These results overall suggested that the increasing morpheme frequency promoted the effect of underlying tonal representation, as in both sandhi constructions, rejecting the underlying tone became more effortful than accepting it whereas rejecting the surface tone became easier than rejecting the underlying tone. The morpheme frequency effect, on the other hand, did not show significant difference between the decisions “yes-surface” and “no-surface” (*p* > 0.05). It indicated that the increasing frequency effect at the morphemic level did not make it easier to accept the surface tone than reject it (neither the other way around).

**Figure 5:**
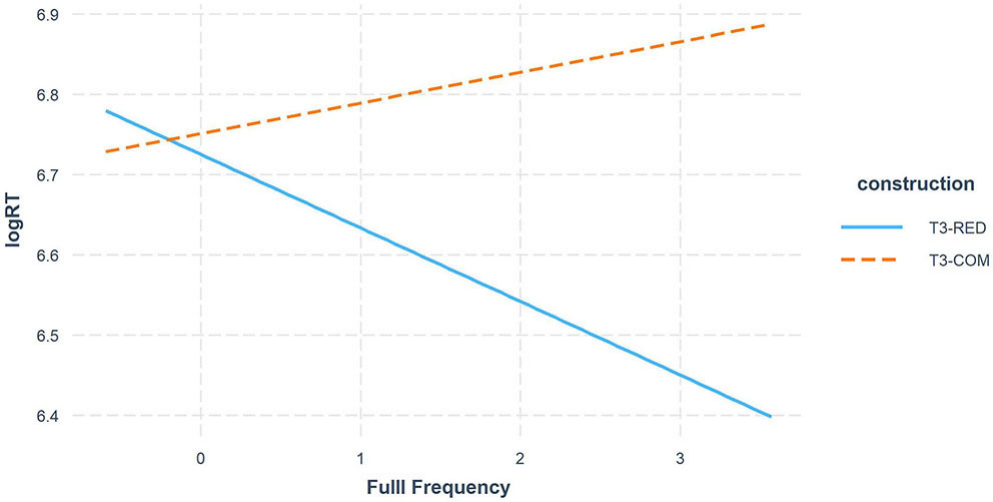
Full Frequency effect in T3-RED vs. T3-COM comparison.

Table 6 also showed a distinct full frequency effect on the T3 sandhi processing between the two morphological constructions (Figure 5), suggesting an inhibitory full frequency effect in T3-COM. More specifically, participants reacted more slowly to sandhi compound words with higher lexical frequency, regardless of which decision they made or which audio tone they heard. This inhibitory lexical frequency effect might seem to contrast with previous findings where high frequency words were identified faster than low frequency words (e.g., Marslen-Wilson, 1987; Goldinger et al., 1989). However, while priming studies focus on spoken word recognition, our present study deals with the explicit tone-morpheme mapping, where participants made matching decisions regarding whether the audio syllable matched the visual morpheme or not. On one hand, both response types and RTs results confirmed the dominant role of the underlying T3 representation over the surface T2; on the other hand, the initial morpheme of a sandhi compound is always realized as a T2 on the surface. As word frequency increases, the initial morpheme is more often realized as a sandhi T2 in the mental lexicon. When participants heard a T3 audio, they thus had to overcome stronger competition from the surface tone in the tonal mapping. Similarly, upon hearing a T2 audio, although the surface representation is promoted by high lexical frequency, the underlying tone still imposed a dominant influence on the tonal mapping. Taken together, the increasing lexical frequency gave rise to a more salient surface tone competitor in sandhi compound, triggering latency in the sandhi processing. In contrast, T2 saliency was not observed in sandhi reduplication since the processing of T3 sandhi was not inhibited by the increasing full-form frequency. That is, participants consistently parsed the sandhi syllable as a T3 rather than a T2 in the reduplicated construction.

In summary, our results showed that in both sandhi compounds and sandhi reduplication, participants were more inclined to map the sandhi syllable to its underlying T3 rather than surface T2. Despite the dominant underlying tonal representation in both constructions, the role of surface tone was still more salient in compounds than in reduplication. Moreover, we found that the frequency effect at the morphemic level showed different patterns across decision types. An inhibitory full-form frequency effect was found in the sandhi compound construction only. The higher morpheme frequency effect promoted the underlying tone by making the rejection of the T3 harder, and the higher lexical frequency made the surface tone a stronger competitor to the underlying tone, thus causing an overall inhibition on tonal mapping.

## Discussion

The current study investigated how the Mandarin T3 sandhi derived by two morphological processes—reduplication and compounding—are represented and processed. We presented findings of a *cross-modal syllable-morpheme matching* experiment, in which participants judged whether the audio stimuli matched the visual morpheme. The results presented here shed light on the morphology-phonology interface in Mandarin Chinese and its influence on T3 sandhi processing. First, while the underlying tone was equally available in sandhi and non-sandhi reduplication, it was more effortful to access in the sandhi construction due to tonal competition. Second, morphological differences influence the processing of T3 sandhi. The underlying and the surface representations are both activated and engage in a competition under the modulation of frequency effects in the compounds, but the surface representation is relatively weakly activated in sandhi reduplication and serves as a weaker competitor of the underlying tone. The distinct processing patterns of T3 sandhi further provide insight into how Mandarin compounds and verb reduplications are represented in the mental lexicon.

### Processing Sandhi in Different Morphological Processes

In both sandhi compounds and reduplications, we found that the underlying representation was strongly activated and can be mapped to the target morpheme. This result is consistent with the findings in Zhou and Marslen-Wilson (1997), Chien et al. (2016) and Meng et al. (2021), where the pre-activation of a T3 prime facilitated the recognition of sandhi targets. Our data support the canonical representation view that the sandhi syllable, whether derived by compounding or reduplication, is represented as its underlying form in the mental lexicon. The strong role of the underlying representation has also been corroborated by previous priming studies on words that involve phonological alternation (Lahiri et al., 1990; Gaskell and Marslen-Wilson, 1996; Gow, 2001, 2002; Luce et al., 2001). Interestingly, we found that the strong accessibility of the underlying representation extends to a rather opaque phonological context—Mandarin verb reduplication where T3 sandhi and tone neutralization jointly induces an opaque [T2+T0] pattern in the speech output. Presumably, the lack of the sandhi context on the surface should make it harder for participants to access the underlying tone. However, this opacity did not keep native speakers from making phonological inferences on the sandhi-surfaced initial syllable, as the underlying tones are equally accessible in the opaque (e.g., T3-RED) and transparent (e.g., T2-RED and T3-COM) structures. While the underlying representation plays a strong role across constructions, the accessibility of the surface representation is different in the two morphological processes—it is more accessible in compounds than in reduplication.

Let's now put together the findings of several studies, including Zhou and Marslen-Wilson (1997), Chien et al. (2016), Meng et al. (2021), and ours to understand how tone sandhi is processed in lexical compounds. Recall that Chien et al. (2016) found no priming effects between T2 primes and T3 sandhi words in an auditory-auditory priming experiment. Both Zhou and Marslen-Wilson (1997) and Meng et al. (2021) found that T2 primes can affect the recognition of sandhi words, despite that the priming effect was inhibitory in the former and but facilitatory in the latter. Our study also found the surface tone T2, in addition to the underlying T3, is associated with the sandhi syllable of a compound word.

Considering these findings, we suspect the diverse findings may be due to task differences. First of all, Chien et al. (2016) adopted an audio-audio priming experiment with an SOA of 250ms while Meng et al. (2021) adopted a cross-modal audio-visual priming task with an SOA of 0ms. The differences between these two studies suggest that accessing a visually presented sandhi compound word can be facilitated by hearing the same sandhi syllable in its surface/output form at the time when the visual word appears. The delay of just 250ms and the audio presentation mode may already cause the priming effect to disappear. Zhou and Marslen-Wilson (1997), on the other hand, used primes that are made of disyllabic (compound) words and found a homophonic inhibitory effect between a T2 prime and a sandhi word. These results suggest that a T2 activates both canonical T2-initial words and T3 sandhi words, thus creating competition in lexical access. Overall, Zhou and Marslen-Wilson (1997) pointed toward competition effects between words that share initial syllables even when these syllables are T3 underlyingly but T2 on the surface.

Our results complemented previous findings by demonstrating the relation between surface tone representations, underlying representations and frequency effects in different constructions. The saliency of the surface T2 was modulated by the whole-word frequency; its representation was more accessible when the word frequency increases. The discovery of the surface T2 effect can also be partially attributed to the research paradigm that we used in the current study. Similar to the design of Meng et al. (2021), we also employed a cross-modal paradigm where an audio input was matched against a visual word (though in our case, the target words were presented in sentences), with an SOA of 0 ms. Our task directly taps into the auditory representations associated with sandhi syllables, thus encouraging a more deliberate decision based on phonological representations. Taken together, our results support the canonical representation view as well as recognize the role of the surface T2 in the lexical access for sandhi-involved compounds.

Disyllabic verb reduplication, on the other hand, exhibits a distinct processing pattern from lexical compounds. Our results show that while the underlying tone representation was strongly activated, the surface tone representation was relatively weak and less likely to be accepted. This pattern was not modulated by any frequency effects. In the verb reduplication that involves T3 sandhi, the base morpheme is underlyingly T3 and realized as a sandhi T2 in the reduplicated full form. The dominant role of the underlying tone over the surface tone indicates that the morphemic representation of these reduplicated monomorphemic verbs is more accessible than the disyllabic full form. This pattern also suggests that native speakers parse the verb reduplication as a reduplicated form of the monosyllabic morpheme that links with the underlying tone representation in the lexicon. Notwithstanding the ceiling accuracy on mapping the sandhi syllable to its underlying tone, participants were slowed down by the co-activated surface T2, as opposed to in the non-sandhi construction. This sandhi-triggered latency indicates that the competition between the underlying and surface tone still exists in sandhi reduplication. Therefore, we confirm the canonical representation view in the processing of sandhi verb reduplication, and that the surface tone exerts a less important role in the processing of verb reduplication than in sandhi compounds.

As pointed out by one of the reviewers, our results that both T2 and T3 are active during sandhi processing may also support representation views other than the canonical and surface accounts. Evidence supporting these alternative accounts mainly comes from electrophysiological experiments using an oddball paradigm. In this particular paradigm, a repetition of audio stimulus (standard) is played to listeners, with a deviant occurring at unexpected positions occasionally. Upon hearing the oddball stimuli, deviation from the standard yields a mismatch negativity (MMN) response, and the amplitude of the MMNs can be modulated by acoustic-phonetic information (e.g., fundamental frequency) as well as phonological differences (e.g., features such as [CORONAL] and [LABIAL]). For example, the amplitude of the MMNs is smaller if the standard is prototypical, i.e., underspecified, and the deviant is atypical, i.e., fully specified (Eulitz and Lahiri, 2004; Näätänen et al., 2007; Cornell et al., 2013). Using the oddball paradigm to probe the representation of Mandarin tone that involves phonological alternation, Li and Chen (2015) constructed four standard/deviant conditions: T1/T3, T3/T1, T2/T3, T3/T2. They found asymmetric MMN effects between T2/T3 and T3/T2 but not between T1/T3 and T3/T1. The MMN amplitude was smaller when T3 is the standard and T2 the deviant, as opposed to the reversed condition, leading them to propose that T3 is represented in both its canonical form and its allophonic variant form (i.e., sandhi tone) in the mental lexicon. These results support the multi-variant account for Mandarin T3 representation, as triggered by the influence of tone sandhi. Politzer-Ahles et al. (2016) further examined Mandarin tonal representation by comparing the MMN effects among all possible combinations of the four tones. Their results showed constant asymmetric MMN effects between the conditions that involved a T3—the MMNs were always smaller whenever T3 was a standard whereas always larger whenever T3 was used as a deviant. Politzer-Ahles et al. (2016) thus concluded that Mandarin T3 is always underspecified, compared to the other three Mandarin tones.

Both Li and Chen (2015) and Politzer-Ahles et al. (2016) used monosyllabic audio and supported the representation views other than the canonical or the surface account. However, since T3 sandhi is a phonological alternation that occurs at the multisyllabic lexical level, it remains less clear whether these results obtained by using the pre-attentive EEG measure can account for the representation of lexical sequences. Chien et al. (2020), to our knowledge, is the only study that used the oddball paradigm to probe the representation of disyllabic T3 sandhi words. In their study, four types of standard stimuli were constructed: a disyllabic T2-initial word /T2+T4/, a disyllabic T3-initial word /T3+T4/, a disyllabic sandhi word /T3+T3/ → [T2+T3], and a mixture of disyllabic sandhi and T3-initial words. The deviant was always a monosyllabic T2 word. Their results showed MMN effects in both the T2 and the T3 condition but not in the sandhi or the mixed condition. According to Chien et al. (2020), the different MMN effects between the conditions T2+T4/T2 (standard/deviant) and T3+T3/T2 (standard/deviant) suggested that distinct neural mechanisms were required between processing the T2 standard words and the sandhi standard words. Moreover, Chien et al. (2020) argued that the lack of the MMN effect in the sandhi condition could have two possible interpretations: (1) that the sandhi standard was not parsed as either a T2 or a T3 word; otherwise we should expect the similar MMN effects as observed in the T2 and the T3 word condition, or (2) that participants productively “rewrote” the initial sandhi syllable as well as the deviant T2 to an underlying T3, thereby no mismatched response was elicited in the sandhi standard condition. These two explanations proposed by Chien et al. (2020) did not differentiate the underspecified and the canonical representation views specifically. Therefore, their results did not provide direct support for the underspecification of sandhi syllables in the mental lexicon.

There are also theoretical issues that make the underspecified view inherently problematic. First, Mandarin tones distinguish lexical meanings and reduces ambiguity during word recognition; therefore, all lexical tones should be specified in Mandarin Chinese. Also noted in Zhou and Marslen-Wilson (1997), since all T3 morphemes can potentially undergo T3 sandhi, underspecifying the tonal information would make lexical access inefficient and involve greater competition. Second, underspecified phonemes usually have default or unmarked features, e.g., coronal place of articulation, whereas Mandarin T3, with the falling-rising pitch contour, is usually considered the most complex and the least frequent tone among the four tonal categories (Zhang and Lai, 2010). Third, underspecified feature often assimilates to a specified one within phonological context, e.g., the underspecified [CORONAL] feature assimilates to the neighboring [LABIAL] feature (/*i**n*-*b**alance*/ → [*i**m*-*b**alance*]). Following this logic, the underspecified Mandarin T3 would assimilate to a more specified tonal feature. However, Mandarin T3 sandhi is clearly a process of dissimilation where the first T3 changes to a T2 when followed by another T3. Therefore, the claim that Mandarin T3 is underspecified cannot fully account for the T3 sandhi rule occurring at the lexical level.

Similar issues also exist for the multi-variant representation view. As proposed by Li and Chen (2015), Mandarin T3 syllable is stored both as its canonical T3 and allophonic sandhi tone. With respect to the representation of disyllabic lexical word, this view should predict that both T3 and T2 can be mapped onto T3-initial words, including both non-sandhi T3 words and T3 sandhi words. However, four experiments conducted in Meng et al. (2021) showed that the representation of T3-initial words may not be fully accounted for by this view. For instance, although both T3 and T2 primes were found to facilitate the recognition of sandhi target/mediator (discussed in section Tone 3 Sandhi Processing in this paper), only T3 prime could activate the non-sandhi T3-initial word (e.g., only *fan*3 primes *fan-she* /T3+T4/ “reflection”) and the non-sandhi mediator (e.g., only *nao*3 primes *tou-bu* /T2+T4/ “head”). These results indicate that both T3 and T2 can activate T3 words *but only within a sandhi context*, and that the multi-variant monosyllabic T3 representation may not be directly relevant to lexical representations.

Back to our current study, the underspecified or multi-variant representation view should predict equal activation between the underlying T3 and the surface T2, with the two tones being mapped to the sandhi syllable with similar percentages and effort. However, this prediction was not supported by our data—the underlying T3 still exhibited a dominant role over the surface T2, and the latter also showed unequal activation between compounds and reduplication. Taken together, the underspecification view and the multi-variant view still require further examination in terms of the representation of Mandarin tones, as well as that of disyllabic sandhi sequences.

### Morphological Representation Models for Compounding and Reduplication

**Figure 6:**
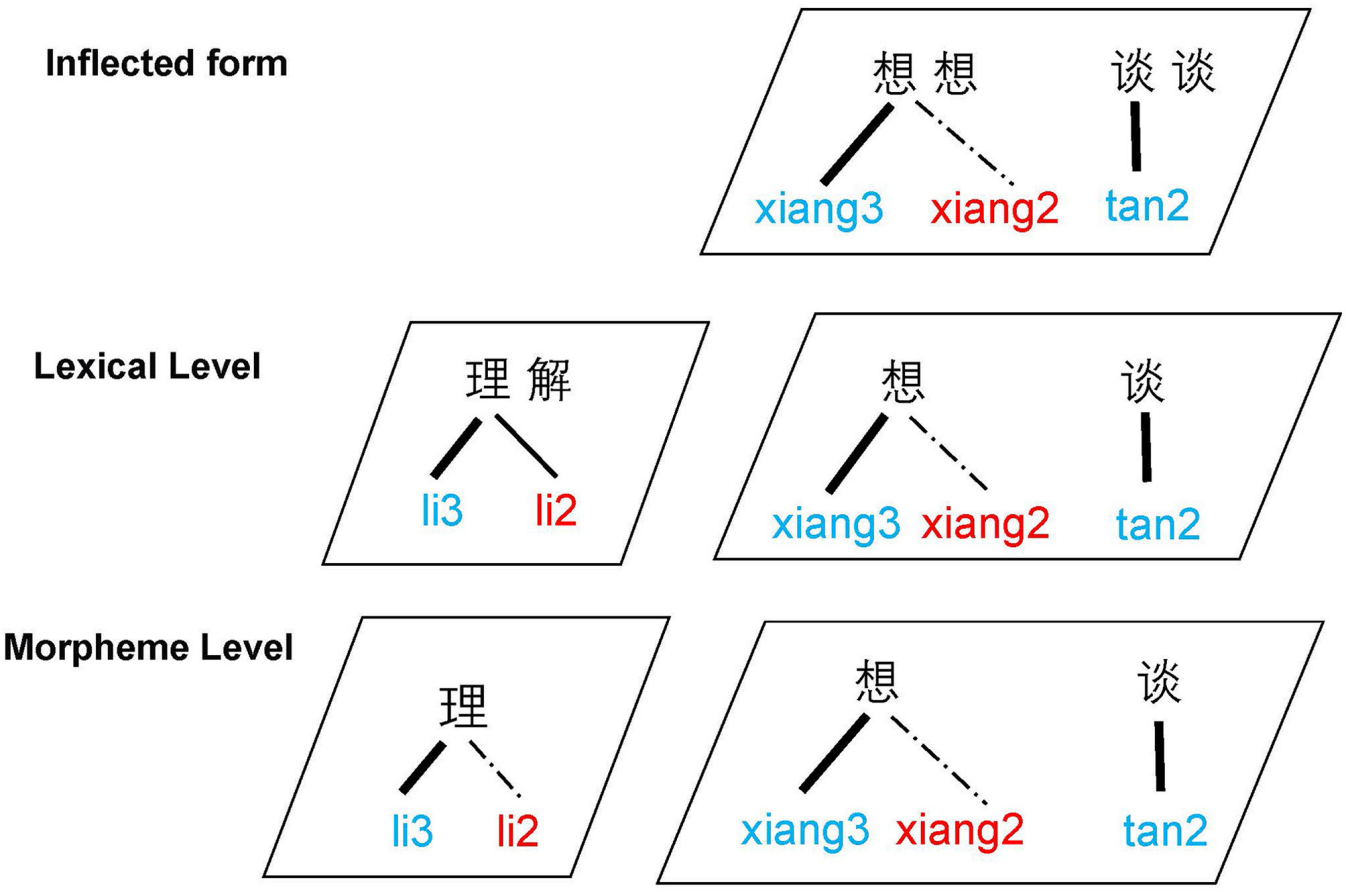
Multi-level representation model for tonal processing.

To understand the role of morphological structure (i.e., reduplication and compounding) in processing T3 sandhi, we propose a *Multi-level Representation Model for Tonal Processing* in Mandarin Chinese (Figure 6), which was inspired by the *Multi-level Cluster Representation Model* (Zhou and Marslen-Wilson, 1994, 1995). Our model links the underlying and surface tone representations to different morphological levels. In Figure 6, phonological forms (syllables with tones) are presented with pinyin-number combinations, in which the underlying representations are coded in blue and surface representations in red. The styles of connecting lines demonstrate the strength of tonal accessibilities in each morphological level—solid lines indicate stronger associations between morpheme and tonal forms whereas dashed lines indicate weaker connections.

For complex words that involve phonological alternations like T3 sandhi, the underlying and the surface tones are realized at different morphological levels. Take the disyllabic sandhi compound *li-jie* /T3+T3/ → [T2+T3] as an example; for the sandhi morpheme *li* /T3/, the canonical T3 links to the morphemic level while the T2 surfaces at the lexical level. In our model, the sandhi surfaced T2 is also linked to the morpheme as an alternative tonal form, posing a weak influence at the morphemic level, whereas the role of surface tone becomes greater at the word-level representation. The availability of both T3 and T2 during sandhi processing indicates that both the morpheme and the word level are actively represented in the mental lexicon and they compete with each other during lexical access, accounting for the inhibitory word frequency effect. Notice that the thickness of lines illustrate the different strengths of association between the T3 and T2. Based on our results, T3 is predominantly activated over T2 in sandhi compounds. This representation is similar to the two-layer lexical access model proposed by Zhou and Marslen-Wilson (1994, 1995), which postulates the combination of morphemic and whole-word representations in the mental lexicon.

The T3-inflected verb reduplication, on the other hand, contains a base verb and a reduplicant in its morphological structure. Similar to sandhi compounds, the base morpheme is represented as a T3 at the morphemic level with an alternative T2 linked. It corroborates with our results in Reduplication vs. Compounding: T3-RED vs. T3-COM that the morpheme frequency exerts similar effects in sandhi compounds and sandhi reduplication. Unlike compound words, reduplication is still represented as its base morpheme at the full-form lexical level where the surface T2 serves as a relatively weaker tonal competitor as opposed to that in sandhi compounds. The base morpheme is phonetically realized as a sandhi T2 post-lexically at the reduplicated output, which can be taken as a level higher than the full lexical form. The ceiling accuracy for the underlying tone suggests that the level of base morpheme is strong for the representation of reduplication. The surface T2, however, was less available as opposed to the underlying T3, providing evidence that native speakers are more inclined to decompose the reduplicated full form (associated with T2) into its base morpheme. As a result, the underlying T3, which is associated with the morpheme/word level, is readily accessible in tonal processing, whereas the sandhi surfaced T2 is only indirectly linked to the morpheme/word. Our findings suggest that verb reduplication in Mandarin is represented and accessed through a monomorphemic T3 word (i.e., *xiang* /T3/ “to think”) in the mental lexicon. On the other hand, for the disyllabic verb reduplication inflected from a T2 base morpheme, the tonal representations associated with the morpheme and the full form are identical—which is T2 across all levels. Therefore, accessing the underlying T2 in these reduplicated forms is relatively easy as it does not involve competition between an underlying tone and a sandhi surfaced tone.

During the online processing for verb reduplication, both the sandhi and non-sandhi, the reduplicated [T2+T0] full form is accessed through its monomorphemic base (e.g., *tan* /T2/ “to talk” and *xiang* /T3/ “to think”). Although verb reduplication is represented as two characters orthographically, only the base morpheme is accessed during lexical access, which is similar to the “affix-stripping” operation (Andrews, 1986; Rastle et al., 2004; Longtin and Meunier, 2005; McCormick et al., 2009; Beyersmann et al., 2011). Our results corroborate that verb reduplication is a different morphological structure from compounding, in that the second constituent likely serves as an affix to the base morpheme and that the whole reduplicated form is not stored as a lexical entry (Li and Sui, 2009; Sui, 2018).

Using the processing of T3 sandhi as a window, the current study provides evidence for the morphological representations of Mandarin Chinese lexicon. Our data support the two-layer representation for processing Mandarin compounds (Zhou and Marslen-Wilson, 1994, 1995), as both morphemic and whole-word representations are accessed and these two layers may engage in competition during lexical processing. The processing pattern for disyllabic verb reduplication, in contrast, is more appropriately accounted for by the morpheme-based representation (Caramazza et al., 1988; Zhang and Peng, 1992), in which the head morpheme as well as its associated tone exhibit greater strength. The disyllabic reduplicated form is not directly retrievable from the lexicon and requires active application of the phonological alternation at the inflectional level. While previous studies mostly focused on T3 sandhi presented in isolated compound words, the present study provided new data targeting sandhi expressions in two distinct structures—compounding and reduplications. It is also worth noting that, to better motivate reduplicated forms, our study presented the targeted expressions in sentential contexts.

### Limitations and Future Directions

We acknowledge some limitations of this study. First, the audio stimuli and target morphemes (in both target and filler trials) only minimally contrasted in tones, not in segments. Although we did not explicitly instruct the participants to do so, such minimal pairings might cause them to pay more attention to the tonal information; as a result, they may become more aware of the difference between T3 and T2. Second, the participants that we recruited in the current study have diverse dialectal backgrounds. The different tonal systems in Chinese dialects could insert a potential influence on the mapping results. Third, we only selected coordinative verb as the targeted compound stimuli, in which both constituents can serve as heads. It still remains unknown whether the results of the current study can extend to the lexical compounds of other structures (e.g., modifier-noun), the sequences containing consecutive T3s, and the T3 sandhi that applies across word boundaries. Future research is needed to further examine the processing of tone sandhi within different types of morphologically complex units.

## Conclusion

In summary, the present study investigated the representation and processing for the Mandarin T3 sandhi sequences derived from two morphological processes—verb reduplication and compounding. We found that the underlying tone exerts a dominant influence on both construction types, whereas the surface tone is more accessible in compounds and is easy to bypass in reduplication. Our study has implications for the interface between the phonological and morphological representations. For lexical compounds, both the whole words and the morphemes are salient levels for morphological representation, and the tonal representations (T2 and T3, respectively) associated with these two levels are activated and compete with each other during sandhi processing. In contrast, the monomorphemic base, rather than the reduplicated full form, is stored in the lexical entry for verb reduplication, and thus the tonal representation associated with the morpheme/word level (e.g., T3) has predominant activation than the derived tone beyond the lexical form (e.g., T2). The current study provides promising evidence that phonological alternations, such as T3 sandhi, can help us understand the differences of morphological structures of Mandarin Chinese, and its importance for understanding speech processing.

## Data Availability Statement

The raw data supporting the conclusions of this article will be made available by the authors, without undue reservation.

## Ethics Statement

The studies involving human participants were reviewed and approved by Indiana University Human Research Protection Program (HRPP). Written informed consent for participation was not required for this study in accordance with the national legislation and the institutional requirements.

## Author Contributions

FG came up with the research ideas, conducted the experiment, ran statistics, and wrote and revised the manuscript. SL built up the experiment, ran statistics, and analyzed and discussed the data with FG. C-JL discussed the research ideas and designs with FG and SL, oversaw the experiment execution, data analysis and interpretation, and revised and edited the manuscript. The study was conducted in the Language and Cognition Laboratory of Indiana University, directed by C-JL, and financially supported by a research grant of C-JL. All authors contributed to the article and approved the submitted version.

## Conflict of Interest

The authors declare that the research was conducted in the absence of any commercial or financial relationships that could be construed as a potential conflict of interest.

## Publisher's Note

All claims expressed in this article are solely those of the authors and do not necessarily represent those of their affiliated organizations, or those of the publisher, the editors and the reviewers. Any product that may be evaluated in this article, or claim that may be made by its manufacturer, is not guaranteed or endorsed by the publisher.

## Footnotes

^1^We were unable to obtain all frequency data from a single corpus because the reduplication stimuli cannot be found in many corpora (including the *Cncorpus*). Considering that the frequency data were retrieved from a different corpus (zhTenTen 2017), we used the standardized frequency (word count/total corpus-size) to keep the results comparable.

^2^The cloze probability task (*n* = 17) and naturalness rating task (*n* = 94) were conducted with two different groups of native Mandarin speakers. The naturalness rating scale was 1–100.

^3^response ~ construction^*^tone + (1+construction^*^tone|Subject) + (1+tone|Item).

^4^response ~ construction^*^tone + (1+tone|Subject) + (1|Item).

^5^logRT ~ construction + MorFreq^*^construction + FullFreq^*^construction + SyllFreq^*^construction + syllable.duration+ rhyme.duration + (1+construction|Subject) + (1|Item).

^6^logRT ~ construction + MorFreq + SyllFreq^*^construction + (1|Subject) + (1|Item).

^7^logRT ~ decision^*^construction + MorFreq^*^decision^*^construction + FullFreq^*^decision^*^construction + SyllFreq^*^decision^*^construction + syllable.duration+ rhyme.duration+ naturalness + (1+decision^*^construction|Subject) + (1+decision|Item).

^8^logRT ~ decision^*^construction + MorFreq^*^decision + FullFreq^*^construction + (1|Subject) + (1|Item).
