# Supplementary material for: Processing Mandarin Tone 3 Sandhi at the Morphosyntactic Interface: Reduplication and Lexical Compounds
Source: Front Psychol. 2021 Aug 26;12:713665. doi: 10.3389/fpsyg.2021.713665 (PMC8427194; doi:10.3389/fpsyg.2021.713665)
Supplement: Supplementary file 1 [file Data_Sheet_1.docx]

**Supplementary Materials**

**Appendix 1 Target words**

Note: All types of frequency are scaled.

| **T3-RED** | | | | | |
| --- | --- | --- | --- | --- | --- |
| **Target** | **Base Tone** | **Morpheme Frequency** | **Full Frequency** | **T2 Syllable Frequency** | **T3 Syllable Frequency** |
| 使使 | shi3 | 3.29564037 | -0.48820029 | 2.14643864 | 1.43264808 |
| 想想 | xiang3 | 4.76188965 | 0.69729435 | -0.41336197 | 0.64868296 |
| 写写 | xie3 | 0.64951424 | -0.41575848 | -0.29217992 | 0.06361561 |
| 找找 | zhao3 | 0.92728075 | -0.35938234 | 3.88530156 | -0.06877343 |
| 喊喊 | han3 | -0.34005267 | -0.48603983 | -0.34005267 | -0.30799538 |
| 摆摆 | bai3 | -0.42452501 | -0.41824556 | -0.24724725 | -0.09862380 |
| 比比 | bi3 | -0.43306684 | -0.45575934 | -0.40341185 | 0.33614430 |
| 躺躺 | tang3 | -0.51124691 | -0.48802337 | -0.30789064 | -0.33397045 |
| 洗洗 | xi3 | -0.20745354 | -0.44776734 | -0.37199040 | -0.32485823 |
| 举举 | ju3 | -0.36997102 | -0.48836360 | -0.34821484 | -0.35146172 |
| 挤挤 | ji3 | -0.46208806 | -0.47704074 | 1.40562564 | -0.34758641 |
| 抹抹 | mo3 | -0.40886741 | -0.48193665 | -0.34989065 | -0.39398541 |
| 瞅瞅 | chou3 | -0.55046144 | -0.46795660 | -0.38832955 | -0.37649414 |
| 炒炒 | chao3 | -0.22503693 | -0.48698567 | -0.38539688 | -0.37911259 |
| 捅捅 | tong3 | -0.51886126 | -0.48770015 | 0.33404953 | -0.35512755 |

| **T3-COM** | | | | | |
| --- | --- | --- | --- | --- | --- |
| **Target** | **Base Tone** | **Morpheme Frequency** | **Full Frequency** | **T2 Syllable Frequency** | **T3 Syllable Frequency** |
| 理解 | li3 jie3 | -0.48750222 | 4.40552434 | -0.26473852 | 1.78865309 |
| 领导 | ling3 dao3 | -0.36560961 | 1.09309773 | -0.31050910 | -0.35837444 |
| 引导 | yin3 dao3 | -0.36117005 | 3.64075833 | -0.38403529 | -0.33093304 |
| 采访 | cai3 fang3 | -0.43884759 | 1.64043963 | 0.42265802 | -0.31658391 |
| 演讲 | yan3 jiang3 | -0.40045092 | -0.17098328 | -0.04897792 | -0.22336695 |
| 保管 | bao3 guan3 | -0.43871381 | -0.02709653 | -0.35146172 | -0.34297793 |
| 选取 | xuan3 qu3 | 0.01502937 | 0.33413265 | -0.38791060 | -0.36685823 |
| 审理 | shen3 li3 | -0.51320586 | 0.13698628 | -0.24201034 | -0.38445424 |
| 指点 | zhi3 dian3 | 0.81229817 | -0.24234316 | 0.02936623 | 1.09539121 |
| 起草 | qi3 cao3 | 0.91275750 | -0.14732708 | 0.78138621 | 0.23936624 |
| 打扫 | da3 sao3 | 1.28030548 | -0.08238394 | -0.23027967 | 0.07178518 |
| 抚养 | fu3 yang3 | -0.55221354 | -0.30332942 | -0.10951657 | -0.36015499 |
| 缓解 | huan3 jie3 | -0.49450331 | 1.46608527 | 1.83452840 | -0.40728716 |
| 反省 | fan3 xing3 | -0.11881371 | -0.28840692 | -0.32213503 | -0.23740186 |
| 主导 | zhu3 dao3 | -0.53264647 | 0.63401154 | -0.32957144 | -0.28998042 |

| **T2-RED** | | | | |
| --- | --- | --- | --- | --- |
| **Target** | **Base Tone** | **Morpheme Frequency** | **Full Frequency** | **T2 Syllable Frequency** |
| 学学 | xue2 | 0.47119919 | -0.42955141 | -0.01430958 |
| 谈谈 | tan2 | -0.05811080 | -0.01519868 | -0.15884824 |
| 提提 | ti2 | -0.03126495 | -0.47173996 | -0.23017493 |
| 读读 | du2 | 0.08055183 | -0.43219500 | -0.26547169 |
| 瞧瞧 | qiao2 | -0.48112226 | -0.36035540 | -0.26253902 |
| 玩玩 | wan2 | 0.63703819 | -0.28857023 | -0.27615498 |
| 摇摇 | yao2 | -0.43850695 | -0.40681383 | -0.33040935 |
| 移移 | yi2 | -0.49622843 | -0.48833638 | -0.27458391 |
| 围围 | wei2 | -0.43519106 | -0.48885013 | -0.34035947 |
| 折折 | zhe2 | -0.48495698 | -0.48797574 | -0.38529214 |
| 闻闻 | wen2 | -0.35456469 | -0.46974280 | -0.29867369 |
| 尝尝 | chang2 | -0.49666631 | -0.34593304 | 0.61212935 |
| 涂涂 | tu2 | -0.44243048 | -0.48424341 | 0.07503207 |
| 砸砸 | za2 | -0.44788408 | -0.48831597 | -0.37785573 |
| 嚼嚼 | jiao2 | -0.52856498 | -0.48813225 | -0.41189564 |

**Carrier Sentences (target trials)**

| **T3-RED** |
| --- |
| 这个 工具 请你 稍微 使使 (Please try this tool a little bit.) |
| 这个 问题 请你 稍微 想想 (Please briefly think about this question.) |
| 这个 反馈 请你 稍微 写写 (Please briefly fill in this questionnaire.) |
| 这篇 文献 请你 稍微 找找 (Please search for this reference for a bit.) |
| 这个 口号 请你 稍微 喊喊 (Please read this slogan for a little bit.) |
| 这个 花瓶 请你 稍微 摆摆 (Please arrange this vase for a little bit.) |
| 这些 价格 请你 稍微 比比 (Please compare these prices for a little bit.) |
| 这个 沙发 请你 稍微 躺躺 (Please lie on this couch for a while.) |
| 这件 衣服 请你 稍微 洗洗 (Please wash this clothe for a bit.) |
| 这对 哑铃 请你 稍微 举举 (Please lift this dumbbell for a while.) |
| 这些 黑头 请你 稍微 挤挤 (Please squeeze these blackheads for a bit.) |
| 这瓶 精油 请你 稍微 抹抹 (Please rinse this bottle of serum for a bit.) |
| 这篇 报道 请你 稍微 瞅瞅 (Please take a brief look at this report.) |
| 这盘 青菜 请你 稍微 炒炒 (Please stir-fry these veggies for a bit.) |
| 这个 炉子 请你 稍微 捅捅 (Please unclog this stove for a little bit.) |

| **T3-COM** |
| --- |
| 这篇 文章 请你 认真 理解 (Please understand this article carefully.) |
| 这些 员工 请你 认真 领导 (Please lead these staff carefully.) |
| 这个 孩子 请你 认真 引导 (Please guide this kid carefully.) |
| 这位 专家 请你 认真 采访 (Please interview this expert carefully.) |
| 这个 专题 请你 认真 演讲 (Please carefully speak about this topic.) |
| 这份 档案 请你 认真 保管 (Please keep this file carefully.) |
| 这些 样本 请你 认真 选取 (Please select the samples carefully.) |
| 这起 案件 请你 认真 审理 (Please process this case carefully.) |
| 这个 学生 请你 认真 指点 (Please tutor this student carefully.) |
| 这份 决议 请你 认真 起草 (Please draft this proposal carefully.) |
| 这个 房间 请你 认真 打扫 (Please clean this room carefully.) |
| 这个 孤儿 请你 认真 抚养 (Please raise this orphan carefully.) |
| 这个 矛盾 请你 认真 缓解 (Please resolve this conflict carefully.) |
| 这个 错误 请你 认真 反省 (Please take a careful reflection on this error.) |
| 这个 项目 请你 认真 主导 (Please lead this project carefully.) |

| **T2-RED** |
| --- |
| 这个 语法 请你 稍微 学学 (Please study this grammar a little bit.) |
| 这件 事情 请你 稍微 谈谈 (Please briefly talk about this issue.) |
| 这袋 水果 请你 稍微 提提 (Please hold this bag of fruit for a while.) |
| 这篇 课文 请你 稍微 读读 (Please read this essay for a little bit.) |
| 这副 画作 请你 稍微 瞧瞧 (Please look at this painting for a while.) |
| 这些 积木 请你 稍微 玩玩 (Please play these toys for a while.) |
| 这面 红旗 请你 稍微 摇摇 (Please shake this flag for a while.) |
| 这把 椅子 请你 稍微 移移 (Please move this chair for a little bit.) |
| 这条 丝巾 请你 稍微 围围 (Please wear this scarf for a while.) |
| 这张 纸片 请你 稍微 折折 (Please fold this paper for a little bit.) |
| 这瓶 香水 请你 稍微 闻闻 (Please smell this perfume for a while.) |
| 这道 甜品 请你 稍微 尝尝 (Please take a small bite of this dessert.) |
| 这瓶 面霜 请你 稍微 涂涂 (Please try this facial cream for a little bit.) |
| 这个 核桃 请你 稍微 砸砸 (Please smash this pecan for a little bit.) |
| 这块 糖果 请你 稍微 嚼嚼 (Please chew this candy bar for a little bit.) |

**Appendix 2 Non-sandhi fillers**

| **Base tone** | **Reduplicated Stimuli** |
| --- | --- |
| **T1** | 说说 shuo-shuo，吃吃 chi-chi，听听 ting-ting，开开 kai-kai，分分 fen-fen，生生 sheng-sheng，穿穿 chuan-chuan，加加 jia-jia，拉拉 la-la，接接 jie-jie，抓抓 zhua-zhua，装装 zhuang-zhuang，收收 shou-shou，拍拍 pai-pai，推推 tui-tui，出出 chu-chu，杀杀 sha-sha |
| **T4** | 去去 qu-qu，看看 kan-kan，用用 yong-yong，做做 zuo-zuo，叫叫 jiao-jiao，问问 wen-wen，笑笑 xiao-xiao，带带 dai-dai，放放 fang-fang，变变 bian-bian，住住 zhu-zhu，望望 wang-wang，站站 zhan-zhan，动动 dong-dong，送送 song-song，唱唱 chang-chang |

| **Base tone** | **Compound Stimuli** |
| --- | --- |
| **T1X** | 需要 xu-yao，开始 kai-shi，说明 shuo-ming，接受 jie-shou，希望 xi-wang，知道 zhi-dao，增加 zeng-jia，加强 jia-qiang，艰巨 jian-ju，帮助 bang-zhu，要求 yao-qiu，发展 fa-zhan，分析 fen-xi，吸收 xi-shou，出现 chu-xian，规定 gui-ding，观察 guan-cha，经营 jing-ying |
| **T2X** | 决定 jue-ding，结合 jie-he，服务 fu-wu，集中 ji-zhong，从事 cong-shi，回答 hui-da，执行 zhi-xing，强调 qiang-diao，承认 cheng-ren，学习 xue-xi，活动 huo-dong，实现 shi-xian，联系 lian-xi，研究 yan-jiu，存在 cun-zai，完成 wan-cheng，培养 pei-yang |
| **T3X** | 使用 shi-yong，改变 gai-bian，采用 cai-yong，组织 zhu-zhi，保护 bao-hu，反映 fan-ying，准备 zhun-bei，考虑 kao-lü，喜欢 xi-huan，产生 chan-sheng，解决 jie-jue，表示 biao-shi，统治 tong-zhi，掌握 zhang-wo，反对 fan-dui，讨论 tao-lun |
| **T4X** | 利用 li-yong，创造 chuang-zao，告诉 gao-su，证明 zheng-ming，注意 zhu-yi，运用 yun-yong，计算 ji-suan，立即 li-ji，进行 jin-xing，控制 kong-zhi，促进 cu-jin，建立 jian-li，处理 chu-li，扩大 kuo-da，确定 que-ding，介绍 jie-shao |

Note: Tonal categories on the second syllable were not manipulated.
